# Missense Mutations Modify the Conformational Ensemble of the *α*-Synuclein Monomer Which Exhibits a Two-Phase Characteristic

**DOI:** 10.3389/fmolb.2021.786123

**Published:** 2021-11-29

**Authors:** Adrien Guzzo, Patrice Delarue, Ana Rojas, Adrien Nicolaï, Gia G. Maisuradze, Patrick Senet

**Affiliations:** ^1^ Laboratoire Interdisciplinaire Carnot de Bourgogne, UMR 6303 CNRS-Université de Bourgogne Franche-Comté, Dijon, France; ^2^ Schrödinger, Inc., New York, NY, United States; ^3^ Baker Laboratory of Chemistry and Chemical Biology, Cornell University, Ithaca, NY, United States

**Keywords:** α-synuclein, amyloid, Parkinson disease, molecular dynamics, dictionary of secondary structure of proteins, CUrvature and Torsion based of Alpha-helix and Beta-sheet Identification, PSEA

## Abstract

*α*-Synuclein is an intrinsically disordered protein occurring in different conformations and prone to aggregate in *β*-sheet structures, which are the hallmark of the Parkinson disease. Missense mutations are associated with familial forms of this neuropathy. How these single amino-acid substitutions modify the conformations of wild-type *α*-synuclein is unclear. Here, using coarse-grained molecular dynamics simulations, we sampled the conformational space of the wild type and mutants (A30P, A53P, and E46K) of *α*-synuclein monomers for an effective time scale of 29.7 ms. To characterize the structures, we developed an algorithm, CUTABI (CUrvature and Torsion based of Alpha-helix and Beta-sheet Identification), to identify residues in the *α*-helix and *β*-sheet from *C^α^*-coordinates. CUTABI was built from the results of the analysis of 14,652 selected protein structures using the Dictionary of Secondary Structure of Proteins (DSSP) algorithm. DSSP results are reproduced with 93% of success for 10 times lower computational cost. A two-dimensional probability density map of *α*-synuclein as a function of the number of residues in the *α*-helix and *β*-sheet is computed for wild-type and mutated proteins from molecular dynamics trajectories. The density of conformational states reveals a two-phase characteristic with a homogeneous phase (state B, *β*-sheets) and a heterogeneous phase (state HB, mixture of *α*-helices and *β*-sheets). The B state represents 40% of the conformations for the wild-type, A30P, and E46K and only 25% for A53T. The density of conformational states of the B state for A53T and A30P mutants differs from the wild-type one. In addition, the mutant A53T has a larger propensity to form helices than the others. These findings indicate that the equilibrium between the different conformations of the *α*-synuclein monomer is modified by the missense mutations in a subtle way. The *α*-helix and *β*-sheet contents are promising order parameters for intrinsically disordered proteins, whereas other structural properties such as average gyration radius, *R*_*g*_, or probability distribution of *R*_*g*_ cannot discriminate significantly the conformational ensembles of the wild type and mutants. When separated in states B and HB, the distributions of *R*_*g*_ are more significantly different, indicating that global structural parameters alone are insufficient to characterize the conformational ensembles of the *α*-synuclein monomer.

## 1 Introduction

*α*-helices and *β*-sheets are the two main secondary structures stabilized by backbone hydrogen bonds in proteins. The propensity of each residue to form an *α*-helix or a *β*-sheet depends on the amino-acid sequence and on the fold (Chou and Fasman, 1974; Smith et al., 1994; Pace and Scholtz, 1998; Bhattacharjee and Biswas, 2010). The formation of long *β*-sheets differs significantly from *α*-helices as the former necessarily involves long-distance contacts in the sequence and depends on the mean curvature of the fold (Koh et al., 2006; Bhattacharjee and Biswas, 2010). The content of these two secondary structure elements is a criterion for classification of protein native structures and characterization of protein folding kinetics and pathways (Konagurthu et al., 2020). In intrinsically disordered proteins (IDPs) (Wright and Dyson, 1999; Uversky et al., 2000; Uversky, 2019; Deiana et al., 2019), *α*-helices and *β*-sheets are metastable secondary structures. As for folded proteins, a natural extension of protein classification of IDP can be based on the content of these two secondary structural elements in a statistical sense. As an IDP has no native state, the relative content of *α*-helices (*α*) and of *β*-sheets (*β*) possibly defines a fingerprint of each conformational state in a two-dimensional (*α*, *β*) propensity map (Ullman et al., 2011). Here, we aim to build such an (*α*, *β*) effective free-energy map for *α*-synuclein (*α*-syn), wild-type (WT), and A30P, A53T, and E46K mutants from first principles by using coarse-grained molecular dynamics (MD) simulations with the UNRES (UNited RESidue) force field (Liwo et al., 2001; Maisuradze et al., 2010).

*α*-syn is a 140 amino-acid protein abundant in the brain (Jakes et al., 1994; Mollenhauer et al., 2008). It plays a central role in the onset of the Parkinson disease (PD) and other neurological disorders, named synucleopathies (Soto, 2003; Chiti and Dobson, 2006; Stefanis, 2012; Chiti and Dobson, 2017; Tanudjojo et al., 2021). In these diseases, *α*-syn is found in high concentration, as filaments, in spherical inclusions (Lewy bodies) located in the neuron cytoplasm (Spillantini et al., 1997; Breydo et al., 2012; Lashuel, 2020; Trinkaus et al., 2021). Familial cases of PD are induced either by an overexpression of WT *α*-syn due to *α*-syn gene triplication or by pathogenic mutations in *α*-syn gene corresponding to single amino-acid substitution, namely, H50Q, G51D, A53E, A30P, A53T, and E46K (Polymeropoulos et al., 1997; Krüger et al., 1998; Zarranz et al., 2004; Fuchs et al., 2008; Appel-Cresswell et al., 2013; Pasanen et al., 2014; Petrucci et al., 2016). These inherited forms of PD have phenotypes that are similar to the sporadic PD, but patients with the A53T mutant exhibit an early onset of PD (Petrucci et al., 2016). The role of *α*-syn in PD development and the mechanisms of its aggregation and of the aggregate propagation are still debatable (Henrich et al., 2020; Sang et al., 2021) and involve *α*-syn-lipid interactions (Galvagnion et al., 2015; Suzuki et al., 2018; Antonschmidt et al., 2021), a liquid-liquid phase transition (Ray et al., 2020), and a secondary nucleation and fibril fragmentation (Cremades et al., 2012; Cascella et al., 2021; Kumari et al., 2021). Moreover, *α*-syn aggregation might disregulate the mitochondrial function, and increasing the level of free radicals and alterations in this pathway may be involved in the pathogenesis of PD (Hsu et al., 2000; Devi et al., 2008).

From a structural point of view, *α*-syn is a chameleon protein (Ullman et al., 2011; Mor et al., 2016): it is disordered (IDP) in solution under physiological conditions, in equilibrium with a minor *α*-helical tetrameric form in the cytoplasm (Bartels et al., 2011; Wang et al., 2011) and *α*-helical when bounded to a cell membrane (Fusco et al., 2018). When incubated under physiological conditions *in vitro*, *α*-syn aggregates into fibrils with polymorphic cross-*β*-sheet conformations, in which a core of *β*-strands is aligned perpendicular to the fibril axis forming extended regular *β*-sheets with different arrangements (Tuttle et al., 2016; Guerrero-Ferreira et al., 2019; Guerrero-Ferreira et al., 2020). In addition to cylindrical fibrils, ribbon aggregates have been also observed (Bousset et al., 2013). The different polymorphs characterized *in vitro* are believed to mimic the *α*-syn filament structures in synucleopathies.

Three main regions were identified in the primary sequence of *α*-syn regarding their role in *α*-syn conformational dynamics and aggregation. The N-terminal region (residues 1–60) contains a number of imperfect repeats, with the consensus motif KTKEGV, strongly similar to that found in the amphipathic helices and responsible for membrane binding (Perrin et al., 2000). High-resolution NMR structures revealed a broken helix featuring two curved *α*-helices of *α*-syn (residues 2—37 and 45—92) bound to micelles (Ulmer et al., 2005), whereas electron paramagnetic resonance characterizations feature an extended helix for *α*-syn bound to a lipid membrane (first 97 residues) (Cheng et al., 2013). In solution, exposure of this N-terminal region to solvents is correlated to aggregation propensity (Stephens et al., 2020). The central hydrophobic region of *α*-syn (residues 61—95), called NAC (non-amyloid component), contains a hydrophobic stretch of 12 residues 71VTGVTAVAQKTV82 necessary for the aggregation (Giasson et al., 2001). Fibrils of the NAC region are also found in plaques of Alzheimer’s disease (Uéda et al., 1993). The flexible acidic C-terminal region regulates fibril formation *in vitro* seeding experiments (Murray et al., 2003) and plays a role in the secondary nucleation process of amyloids via electrostatic interactions with the lysine-rich N-tail (Kumari et al., 2021). Transient, long-range interactions between the negatively charged C-terminus (residues 120—140) with the positively charged N-terminus and NAC (residues 30—100) were observed by paramagnetic relaxation enhancement (PRE) for WT *α*-syn (Dedmon et al., 2005). It is hypothesized that these transient interactions are responsible for the larger compactness of *α*-syn compared to a disordered chain (Dedmon et al., 2005). Structures with long-range contacts between the N-terminal and C-terminal represent about 14% of the conformational ensemble, and in a significant fraction of these structures, residues 68—78 (NAC) are exposed to a solvent, in contrast to the hypothesis that such long-range transient interactions prevent aggregation (Ullman et al., 2011).

Mutations affect the arrangement and growth of the fibrils *in vitro* (Guerrero-Ferreira et al., 2020). Compared to WT, A53T and E46K mutations aggregate faster, whereas A30P aggregates more slowly than WT (Tosatto et al., 2015; Stephens et al., 2020). In addition, WT, A53T, E46K, and A30P differ in the formation of the different early oligomeric moieties (Tosatto et al., 2015). The mutants A30P and A53T show a greater propensity to form non-fibrillar aggregates than WT (Li et al., 2002), and A53T promotes seeded aggregation in human neurons (Tanudjojo et al., 2021). The rate of lipid-induced aggregation and secondary nucleation have been found to differ by multiple orders of magnitude depending on which missense mutation is involved (Flagmeier et al., 2016). NMR studies have shown an increase flexibility of *α*-syn in nanosecond–microsecond time scales and a reduction of contacts between C- and N-terminals in mutants (Bertoncini et al., 2005). All single amino-acid substitutions have thus both a kinetic and structural effect on the formation of oligomeric structures. The purpose of the present theoretical study is the characterization of the differences between the conformational ensembles of monomeric WT and mutants, which may contribute to our understanding of the early steps of the aggregation process in solution.

The majority of monomeric WT *α*-syn conformations have no secondary structures (Ullman et al., 2011). The average helical and *β*-strand contents of WT *α*-syn deduced from restrained MD of *α*-syn fragments and NMR data are about 3 and 11%, respectively (Ullman et al., 2011). The maximum fraction of residues with helical and *β*-sheet secondary structures was found to be 20% (28 residues) and 28% (39 residues), respectively (Ullman et al., 2011). Little is known on how the missense mutations modify the propensity of *α*-syn to form secondary structures. As the *α*-helical/*β*-sheet equilibrium is central to the folding polymorphism of *α*-syn, we decided to characterize the propensities of these two secondary structures in WT, A30P, A53T, and E46K monomers using MD.

Because the huge conformational space of *α*-syn is out of range for all-atom MD simulations in the explicit solvent, we applied coarse-grained UNRES MD (Maisuradze et al., 2010) to sample the structures of WT and the most studied A30P, A53T, and E46K monomers. The effective time scale of UNRES is 3 orders of magnitude larger than the all-atom time scale (Khalili et al., 2005). To ensure the convergence of the conformational sampling of the monomeric states, we applied replica exchange MD (see the Material and Methods section). The total effective time scale of the present simulations is 29.7 ms (72 replicas of 412 *μ*s each) for each protein studied. One of the gold standards to quantify the secondary structure elements of a protein from its structure is the Dictionary of Secondary Structure of Proteins (DSSP) (Kabsch and Sander, 1983; Touw et al., 2015) algorithm based on a simplified model of hydrogen bonds. Application of DSSP to coarse-grained structures simulated by UNRES requires to build a compatible all-atom structure from the *C^α^* coordinates of the UNRES model using reconstruction programs (Feig et al., 2004; Rotkiewicz and Skolnick, 2008). To avoid the high computational cost of all-atom reconstructions from coarse-grained coordinates, we developed here an algorithm which assigns an *α*-helix or a *β*-sheet secondary structure to each residue based on the *C^α^*-*C^α^* distances and on the coarse-grained angles formed by *C^α^*-*C^α^* pseudobonds, which correspond to the local curvature and torsion of the protein main chain (Grassein et al., 2020). The accuracy of the present algorithm, named CUTABI (CUrvature and Torsion based of Alpha helix and Beta-sheet Identification), to quantify the *β*-sheet content of proteins is improved compared to an existing algorithm based on *C^α^* coordinates [P-SEA (Labesse et al., 1997)] and is comparable to the accuracy of DSSP (see the Material and Methods section). For each structure of the conformational ensemble of WT and mutants monomers, the number of residues in *α*-helix or in *β*-sheet was computed with CUTABI. The probability density of this two-dimensional descriptor was computed by using the conformations at 300 and 310 K. Analysis of these maps and of the conformational ensembles of the WT protein and mutants revealed subtle effects of the single amino-acid substitutions which are possibly related to the differences observed in oligomerization between WT and mutants (see the Results and Discussion section).

## 2 Materials and Methods

### 2.1 Coarse-Grained Molecular Dynamics Simulations

Detailed descriptions of the UNRES force field and its parameterization are available in the reference (Liwo et al., 2019) and at http://www.unres.pl. Therefore, it will be only briefly outlined here. In the UNRES force field, a polypeptide chain is represented as a sequence of *C^α^* atoms with united peptide groups located halfway of the virtual *C^α^*-*C^α^* bonds and united side chains (SCs) attached to the *C^α^* atoms. The force field has been derived as the potential of mean force (PMF) of a system of polypeptide chain(s) in the solvent, where all degrees of freedom except the coordinates of the *C^α^* atoms and SC centers have been averaged out. The effective energy function contains local and site–site interactions as well as multibody terms, which have been obtained by decomposing the PMF into factors corresponding to clusters of interactions within and between coarse-grained sites (Liwo et al., 2001). The SC-SC interaction potentials implicitly include the contribution from solvation (Liwo et al., 2001; Maisuradze et al., 2010). The force field was calibrated to reproduce the structure and thermodynamics of small model proteins and applied with success to simulate protein folding (Maisuradze et al., 2010; Zhou et al., 2014; Sieradzan et al., 2021) and large-scale conformational dynamics (Gołaś et al., 2012).

All structures of *α*-syn (WT and mutants) were extracted from replica exchange MD trajectories generated with the UNRES force field. A total of 72 trajectories were computed for each protein: 32 trajectories at 300 K and 8 trajectories at each of the following temperatures, 310 K, 323 K, 337 K, 353 K, and 370 K. Each trajectory was started with two fully unfolded monomers separated by a distance of 25 Å. The Cartesian coordinates of *C^α^* and SC beads were saved every 1,000 integration steps. The integration time step in UNRES is 4.9 fs, corresponding to an effective actual time step of about 4.9 ps. Due to the implicit integration of fast motions, the time scale of UNRES compared to the experimental time scale is indeed accelerated by a factor of 1,000 (Khalili et al., 2005). For each trajectory, the first 4 million steps were discarded. After this relaxation period, 8 consecutive runs of 10 million steps were used for the analysis of each trajectory. Convergence of the conformational ensemble simulated for each protein was monitored by the probability densities of intra-chain and inter-chain contacts and of the radius of gyration. For each trajectory, the convergence was achieved for the last 3 runs of 10 million steps, i.e., for a statistics of 30 million steps (an effective time scale of 147 microseconds) out of 84 million steps (an effective time scale of 412 microseconds). Only structures at 300 and 310 K are reported here as they are close to the physiological temperature. The structures were saved every 1,000 integration steps, leading to about 1 million of converged conformations analyzed for each protein combining the results at 300 and 310 K.

Since the simulations are performed on two monomers, both isolated non-interacting monomer conformations and aggregated monomers were observed in the converged MD trajectories. The analysis and description of the dimeric conformations are out of the scope of the present paper, which is dedicated to isolated monomers, and they will be described elsewhere. The monomeric structures studied in the present work were extracted from the converged replica exchange MD trajectories if they obey the following condition: no residues at a distance smaller than 20 Å from the other monomer. The fraction of monomers out of all the conformers simulated at 300 and 310 K is 55% for WT, 65% for A30P, 50% for A53T, and 48% for E46K. The monomeric state is finally described here by about 1 million of structures of the converged production part of the MD trajectories at 300 and 310 K, representing a sampling on an effective time scale of 4.9 ms.

### 2.2 Curvature and Torsion Based of *α*-Helix and *β*-Sheet Identification: An Algorithm for Secondary Structure Determination Based on C*^α^* Coordinates

Secondary structure elements (SSEs) are important descriptors of the native state of proteins (Konagurthu et al., 2020) and of the conformational ensemble of IDP, as shown in the next section for *α*-syn. The main local structures stabilized by backbone hydrogen bonds are helices (*α*-, 3_10_-, and *π*-helices) and *β*-sheets. Deviations from the canonical definitions of these secondary structures based on Ramanchandran angles are common in the Protein Data Bank. Therefore, a practical and widely used method to assign an SSE to a residue is to apply the DSSP algorithm which is based on the calculation of a simplified energy function describing backbone hydrogen bonds (Kabsch and Sander, 1983). Application of DSSP to a protein structure requires the knowledge of the Cartesian coordinates of all backbone atoms. For structures simulated by coarse-grained force fields, like UNRES, only a subset of these coordinates is known, those of the *C^α^* atoms. Assignment of the SSE based on *C^α^* coordinates can be performed using DSSP but at the extra computational cost of the reconstruction of an all-atom representation of the coarse-grained structure using software like MMTSB (Feig et al., 2004) or PULCHRA (Rotkiewicz and Skolnick, 2008). Alternatively, the SSE can be defined from the *C^α^* coordinates only [P-SEA algorithm (Labesse et al., 1997)]. Here, we have chosen the second faster option and have developed the algorithm CUTABI by analyzing 14,652 experimental structures with less than 40% of sequence identity extracted from the ASTRAL database (Fox et al., 2014; Chandonia et al., 2019). The parameters of CUTABI were adjusted to reproduce the DSSP assignment of helices and *β*-sheets for the ASTRAL database. The agreement between CUTABI and DSSP is 93% for the structures in the database with 10 times (DSSP + PULCHRA) to 30 times (DSSP + MMTSB) less computational cost.

#### 2.2.1 Parameters of Curvature and Torsion Based of *α*-Helix and *β*-Sheet Identification

Assuming a constant virtual bond length between *C^α^* atoms of successive residues, a chain of *N* amino acids is fully characterized by *N* − 3 torsion angles *γ*_*n*_, built from the positions of *C* ^*α*^_*n*−1_, *C* ^*α*^_*n*_, *C* ^*α*^_*n*+1_, and *C* ^*α*^_*n*+2_ with *n* = 2 to *N* − 2; and *N* − 2 bond angles *θ*_*n*_, built from *C* ^*α*^_*n*−1_, *C* ^*α*^_*n*_, and *C* ^*α*^_*n*+1_, with *n* = 2 to *N* − 1. These angles have clear geometrical meanings: they are respectively the discrete version of the local curvature (*θ*_*n*_) and the local torsion (*γ*_*n*_) of the chain formed by the successive *C^α^* − *C^α^* virtual bonds (Grassein et al., 2020). From a mathematical point of view, the local curvature and torsion fully describe the structure of a string and form a complete set of local order parameters for protein folding (Grassein et al., 2020). For proteins, the curvature has a limited range with *θ* varying between 80° and 160°, whereas *γ* can take nearly any value between -180° and +180°. The SSEs correspond statistically to specific areas in the coarse-grained (*γ*, *θ*) maps. Supplementary Figure S1 shows the distribution of (*γ*, *θ*) angles computed by the DSSP algorithm for the 14,652 experimental structures extracted from the ASTRAL database for helix (H + G + I) and *β*-sheet (E) (in brackets the one-letter SSE codes in DSSP). The areas corresponding to helix and *β*-sheet identified by DSSP in the (*γ*, *θ*) map are nicely reproduced by the CUTABI algorithm, as shown in Supplementary Figure S1, using an identification of the SSE based on the coordinates of the *C^α^* atoms as follows.

**FIGURE 1:**
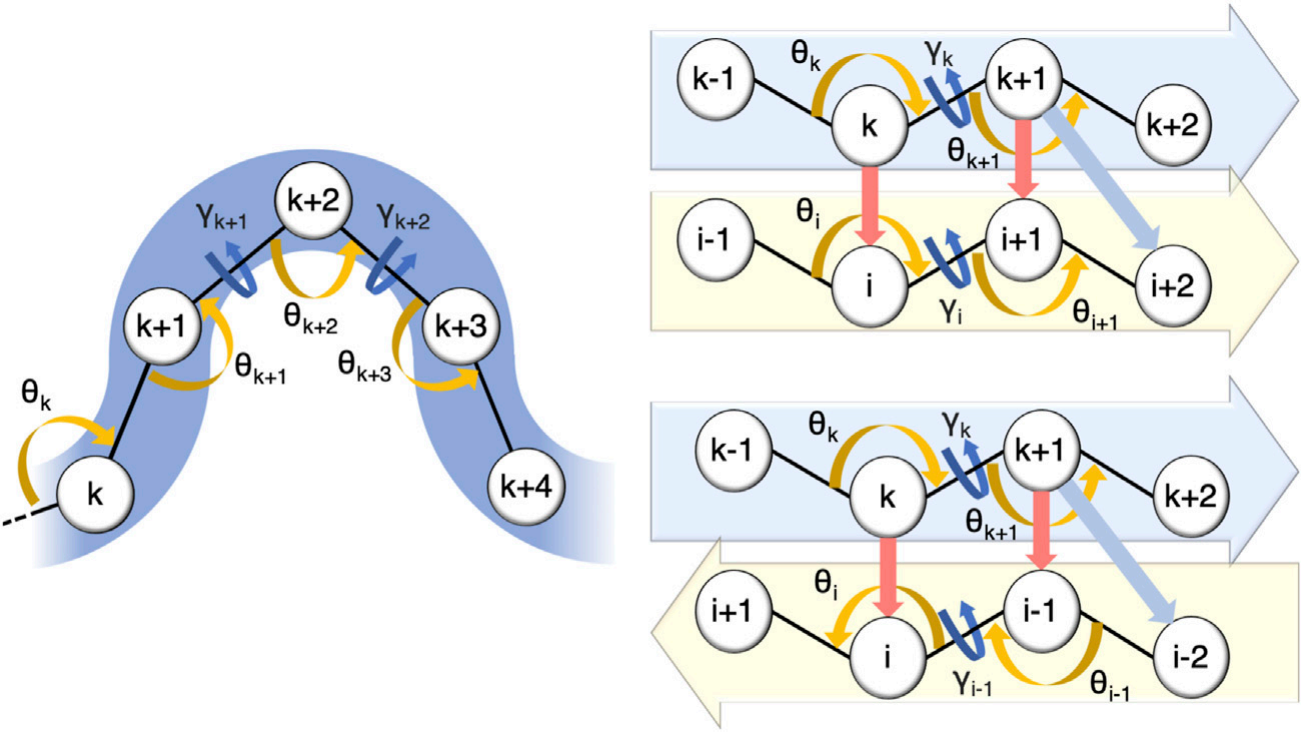
Parameters defining residues pertaining to a helix and to a parallel or to an anti-parallel *β*-sheet in CUTABI (see text). The spheres represent the positions of *C^α^* atoms of the residues. Orange and dark-blue arrows show the *θ* and *γ* angles involved, respectively. For *β*-sheets, red and light-blue arrows point out the distances between residues involved in their definitions.

In CUTABI, the minimal size of a helix is set to 4 residues. Helices with less than 3 residues, such as short 3_10_ helices, are thus not counted. Because helices involve short-range interactions along the amino-acid sequence, the local curvature *θ* and torsion *γ* parameters are sufficient to characterize this SSE. To decide if a set of four residues *k*, *k* + 1, *k* + 2, and *k* + 3 pertains to a helix, a combination of the coarse-grained angles formed by residues (*C^α^* atoms) *k* − 1, *k*, *k* + 1, *k* + 2, *k* + 3, and *k* + 4 is considered, as shown in Figure 1. Residues *k*, *k* + 1, *k* + 2, and *k* + 3 pertain to a helix if the angles *θ*_*k*_, *θ*_*k*+1_, *θ*_*k*+2_, and *θ*_*k*+3_ belong to an interval between 80° and 105° and the angles *γ*_*k*+1_ and *γ*_*k*+2_ belong to an interval between 30° and 80°. A window of 4 residues is slid along the sequence to evaluate the residues pertaining to a helix in each *α*-syn conformation.

In CUTABI, the minimal size of a *β*-strand is set to 2 residues, i.e., a *β*-sheet cannot be smaller than 4 residues. The *β*-strands of 1 residue forming *β*-bridges (B code in DSSP) are thus not considered. To define parallel and anti-parallel *β*-sheets (Figure 1), both local curvature and torsion parameters as well as distances between the two *β*-strands forming the sheet are necessary. If the four residues *k*, *k* + 1, *i*, and *i* + 1 pertain to a parallel *β*-sheet (Figure 1), then the angles *θ*_*k*_, *θ*_*k*+1_, *θ*_*i*_, and *θ*_*i*+1_ must be between 100° and 155° and the angles *γ*_*k*_ and *γ*_*i*_ must be smaller than −80° or larger than 80°. In addition, the distances between *k* and *i* and *k* + 1 and *i* + 1 (Figure 1, red arrows) must be smaller than 5.5 Å, and the distance between *k* + 1 and *i* + 2 (Figure 1, light blue arrows) must be smaller than 6.8 Å. Similar conditions must be met for the anti-parallel *β*-sheet, as shown in Figure 1.

#### 2.2.2 Performance of Curvature and Torsion Based of *α*-Helix and *β*-Sheet Identification Compared to Dictionary of Secondary Structure of Proteins and P-SEA

**FIGURE 2:**
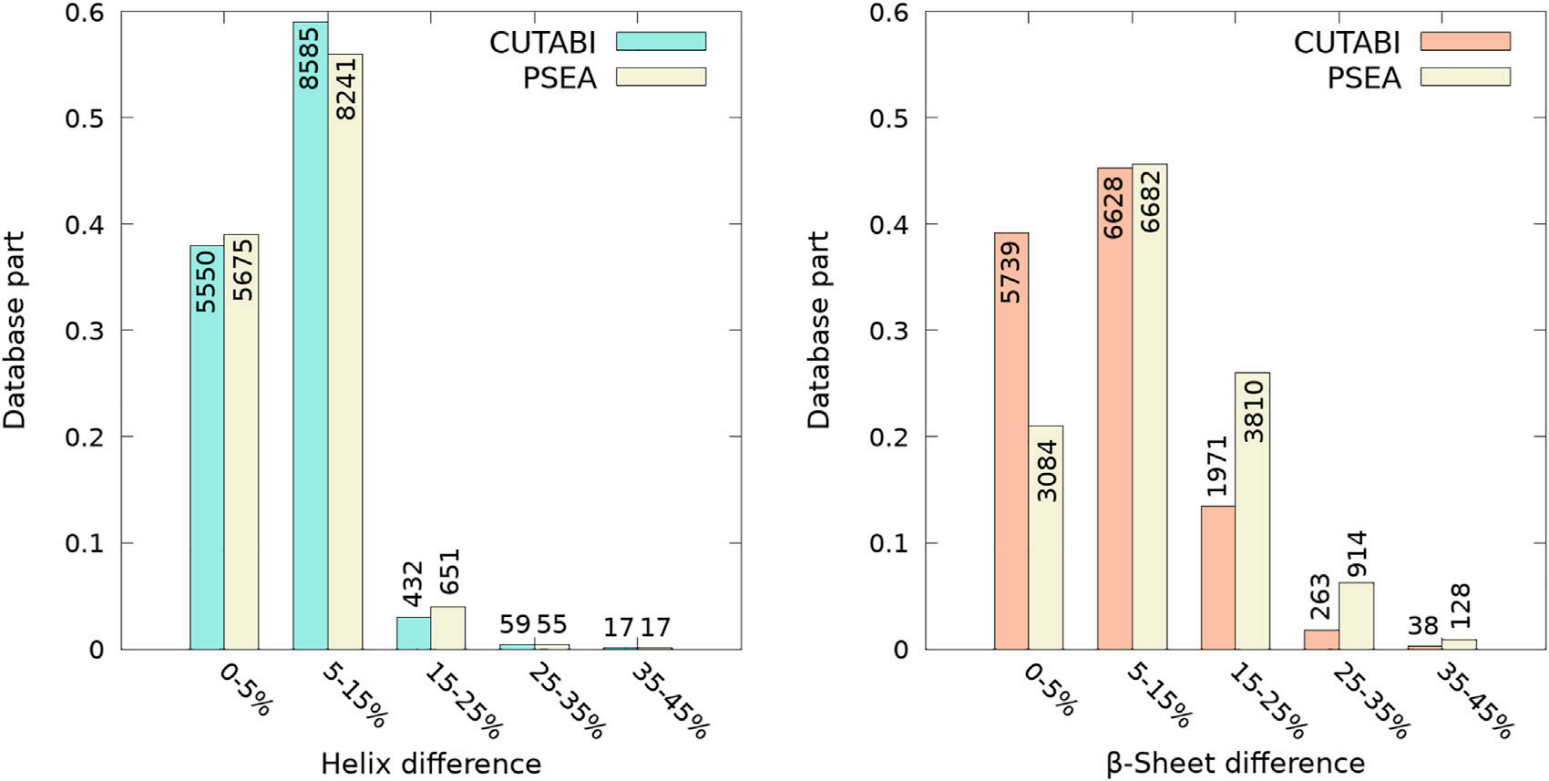
Comparison between the identification of residues pertaining to the helix and to *β*-sheet between the all-atom DSSP algorithm and coarse-grained CUTABI and P-SEA algorithms. Each bar of the histograms represents the number of structures of the ASTRAL database as a function of a range of percentages of the difference between CUTABI (green and orange) and DSSP and between P-SEA (yellow) and DSSP. For each protein, the percentage is calculated as the number of residues having a different secondary structure in the coarse-grained algorithms and DSSP, divided by the protein sequence length.

The SSEs computed with the parameters defined in Figure 1 were compared to the SSEs calculated with DSSP (based on all-atom coordinates) (Kabsch and Sander, 1983) and P-SEA (based on *C^α^* coordinates only) (Labesse et al., 1997). Figure 2 shows the percentage of difference, i.e., the number of residues having an SSE different in the coarse-grained algorithms P-SEA and CUTABI compared to DSSP, divided by the sequence length. For helices, as shown in Figure 2 (left panel), 96% of structures (5,550 + 8,585 = 14,235 structures) do not have more than 15% of residues with an SSE different in CUTABI and in DSSP. The results of P-SEA are similar. Considering DSSP as a gold standard, the CUTABI accuracy is improved compared with P-SEA on the evaluation of residues in the *β*-sheet. As shown in Figure 2 (right panel), 5,739 structures examined (40% of the database) have less than 5% of residues pertaining to an SSE different in CUTABI compared to DSSP. This number is only 3,084 (20% of the database) in P-SEA. In total, assignments of SSE by CUTABI and DSSP agree for 84% of the structures studied (5,739 + 6,628 = 12,367 structures) with a difference compared to DSSP of maximum 15% of the sequence length. The average of the percentage of agreement over all the structures of the database (14,652) for all SSEs between CUTABI and DSSP is 93% for a computational time at least 10 times smaller.

**FIGURE 3:**
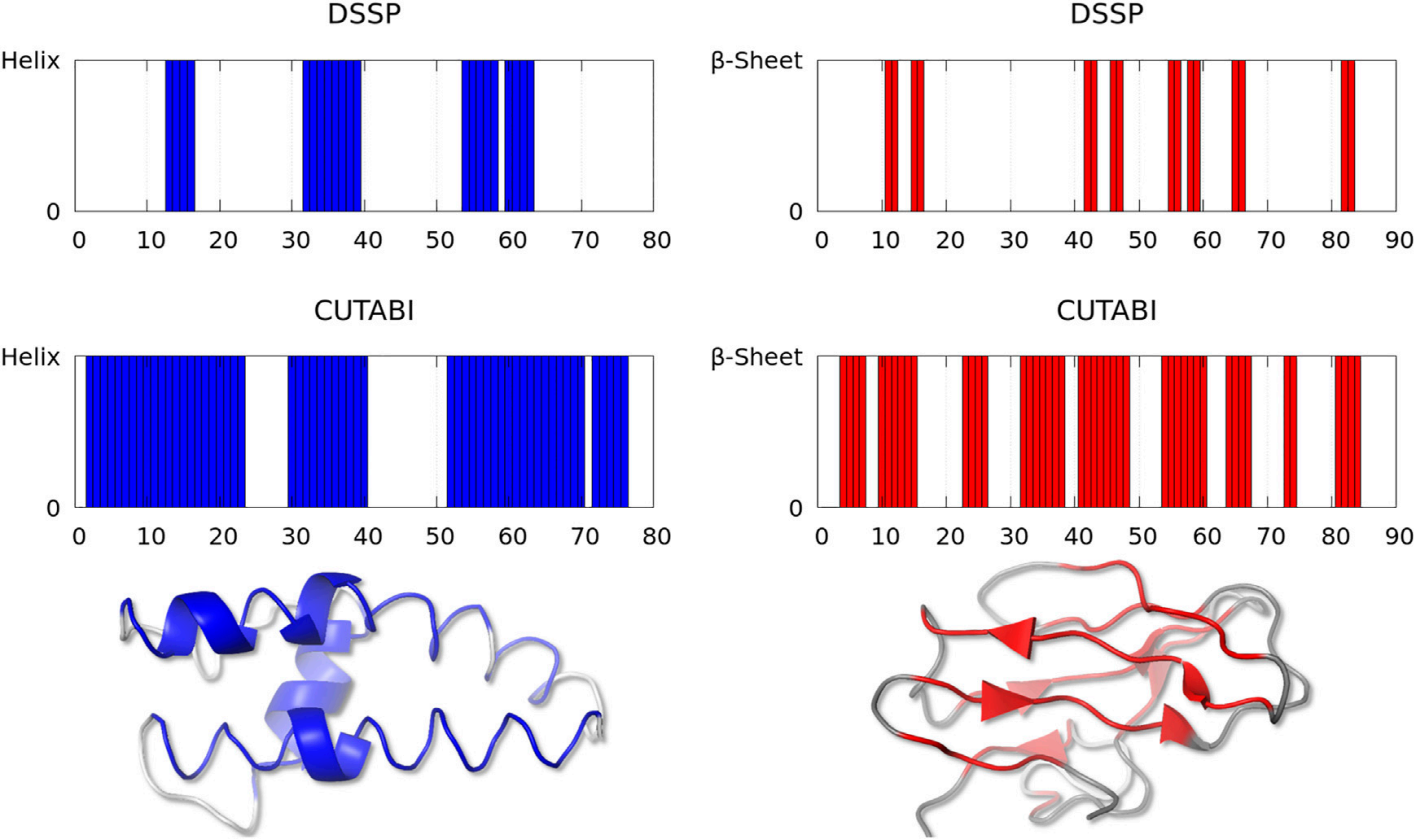
Representative structures with a large discrepancy between the SSE assignment by DSSP and CUTABI for the helix (left, ASTRAL ID: d1lnqa2) and for the *β*-sheet (right, ASTRAL ID: d1eh9a1). In the left (right) panel, each blue (red) bar represents a residue pertaining to the helix (to the *β*-sheet). The 3D main chain is represented with a blue (red) tube for residues pertaining to the helix (to the *β*-sheet) for CUTABI and with a blue (red) cartoon for DSSP. Residues not in the helix or in the *β*-sheet are in gray. The 3D representations were made with the PyMOL software (Schrödinger, 2015).

As shown in Figure 2, one observes a large difference of secondary structure assignments between CUTABI and DSSP for a very small number of proteins. Most structures in this category were measured by X-ray diffraction but with a low resolution (>3Å). In addition, as hydrogen atom positions are not detected in the X-ray (except for ultra-high resolution), the application of DSSP to these structures may be less accurate as DSSP is based on the calculation of hydrogen bond energy. To illustrate the precise origin of this finding, we examined the structures and selected two representative structures with 35% of difference in SSE: one for the helix (Figure 3, left blue panel) and another for the *β*-sheet (Figure 3, right red panel).

Figure 3 (left panel) represents the fragment (from residue 19 to 98) of a calcium-gated potasium channel (PDB ID: 1lnq and ASTRAL ID: d1lnqa2). In this example, CUTABI detects a much larger number of residues in the helix than DSSP. However, the 3D representation shows that although these extra residues missed by DSSP are not in a canonical helix, the overall shape of the main chain is indeed helical.

Figure 3 (right panel) represents the fragment (from residue 1 to 90) of a glycosyltrehalose trehalohydrolase (PDB ID: 1eh9 and ASTRAL ID: d1eh9a1). The number of residues in the *β*-sheet is much larger in CUTABI than in DSSP. As in the case of the helix, the 3D representation indicates that the additional residues in the *β*-sheet in CUTABI are part of a main chain segment with the overall shape of the *β*-sheet, although not canonical, due probably to the low experimental resolution.

## 3 Results

### 3.1 *α*-Helix and *β*-Sheet Propensities of *α*-syn Divide the Conformations Into Two Distinct States

**FIGURE 4:**
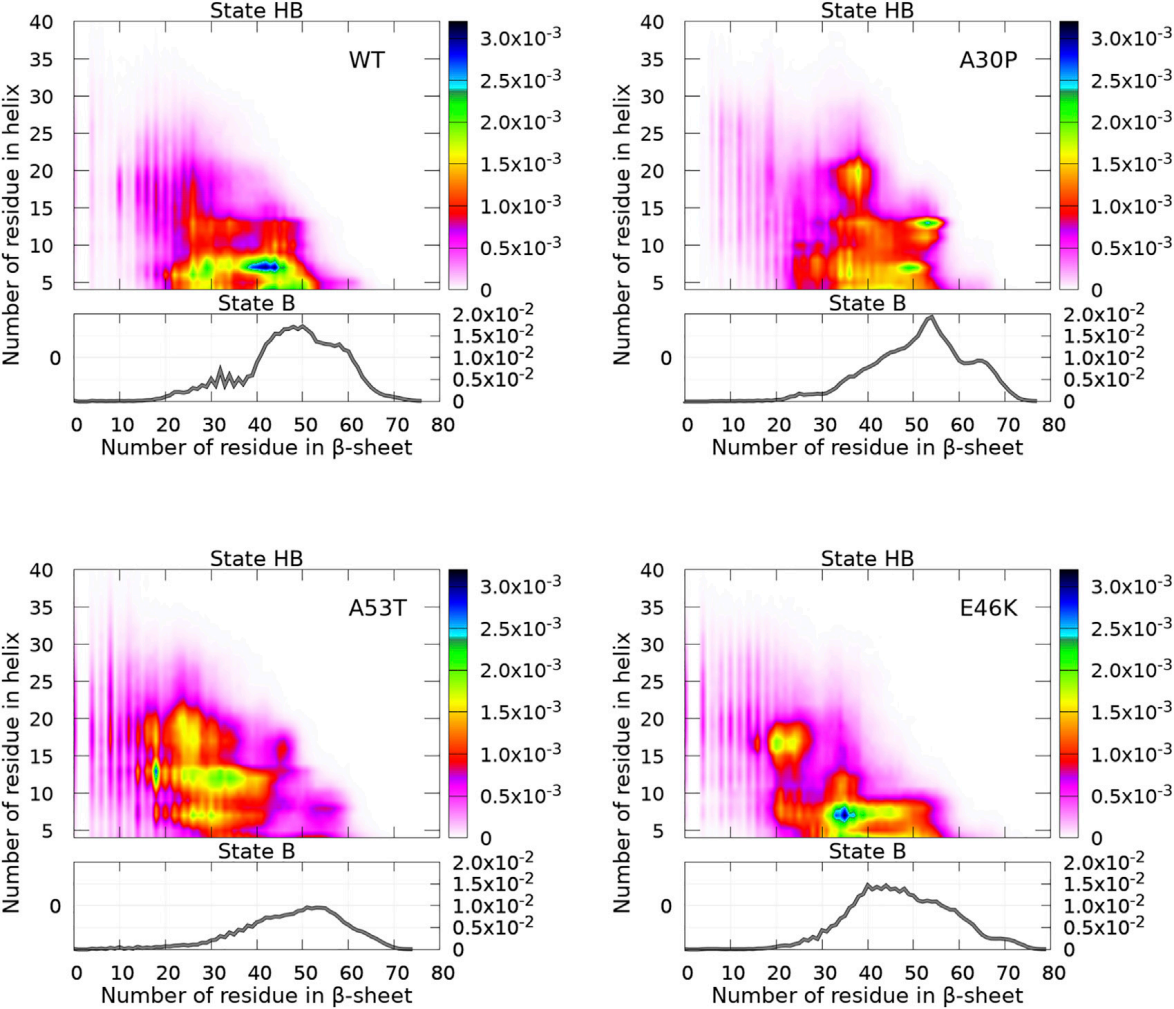
Probability density of the number of residues in the *α*-helix and *β*-sheet for WT and mutants of *α*-syn. The probability density of state B (no helix) is represented by a function (gray) (right vertical axis), and the probability density of state HB is represented by a two-dimensional map (right color bar).

The algorithm CUTABI was applied to the *α*-syn conformations to compute the number of residues in the *α*-helix (*α*) and *β*-sheet (*β*) of each structure for WT and mutants. Each conformation has (*α*, *β*) coordinates. The resulting probability densities in the (*α*, *β*) space are represented in Figure 4. In these maps, only the residues from the N-terminal and NAC regions were considered for the calculations [as can be seen in the next section (Figure 8), the C-terminal region does not contribute to SSE differences between WT and mutants].

A major observation is that the conformations are divided into two distinct states for the N-terminal + NAC region: an ensemble of conformations with no residue in the helix (state B) and the rest of conformations (state HB). The highest probability of observing a conformation in state B is an order of magnitude larger than that of state HB, as can be seen by comparing (the scale of) two-dimensional and one-dimensional plots in Figure 4. In addition, Figure 4 reveals clear differences between WT and mutants.

**FIGURE 5:**
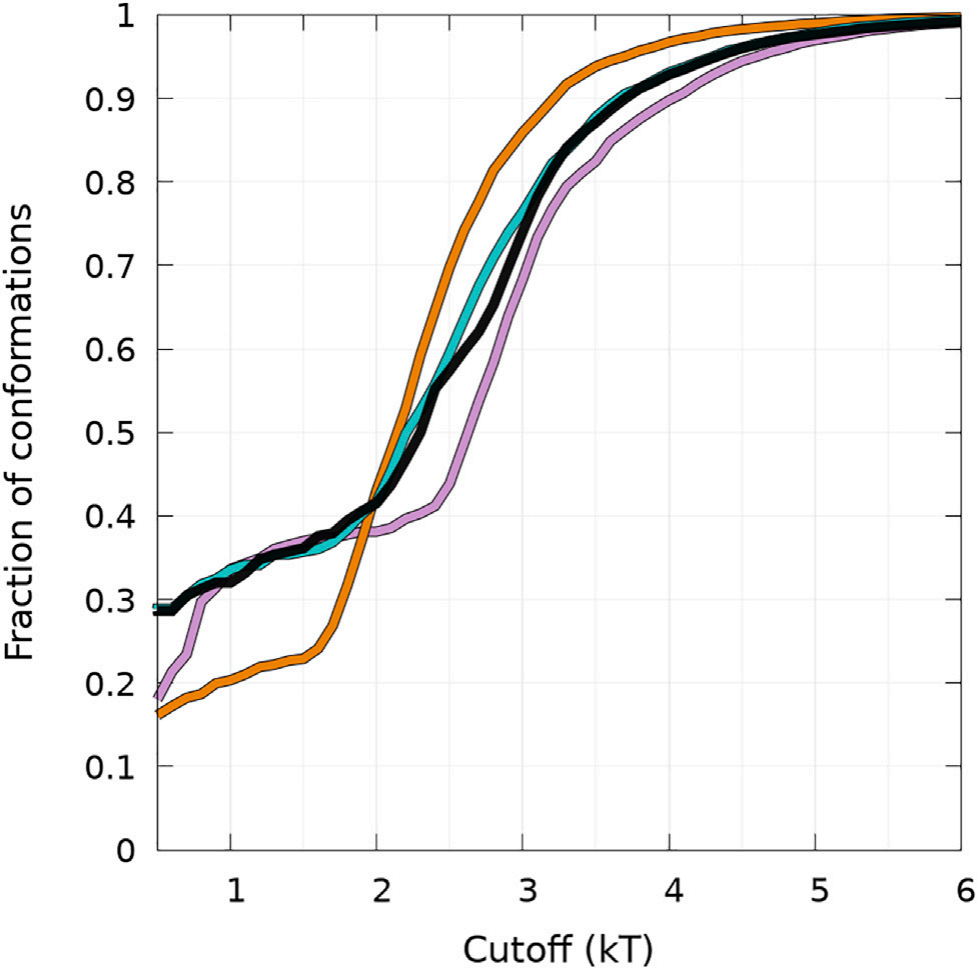
Cumulative fraction of the total number of conformations simulated as a function of a free-energy cutoff (in kT units) from the global minimum of the B state (see text) represented in Figure 4. Curves are for WT (black), A30P (purple), E46K (turquoise), and A53T (orange).

First, we discuss the global differences between states B of WT and mutants (one-dimensional functions in Figure 4). The maximum of the distributions is at 50, 54, 53, and 40 for WT, A30P, A53T, and E46K, respectively. The distribution is sharper for A30P, which has the largest number of conformations with the largest number of residues (between 60 and 70) in the *β*-sheet. Clearly, A53T has the lowest number of conformations in state B. This is even better seen in Figure 5, showing the fraction of conformations within a free-energy difference cutoff from the global minimum of state B for each protein. With *P*_max_ being the maximum of probability at (0, *β*) (in the B state) and *P* being the probability at $\left(\alpha ,\beta \right)\;\left(\alpha \geq 0\right.$ , in the B or HB states), the free-energy cutoff is computed as $-\ln\left(\frac{P_{\max}}{P}\right)$ in *kT* units, where *k* is the Boltzmann constant and *T* is the temperature. Within 1 kT, there is 32% of the conformations found for WT, E46K, and A30P and only 20% for A53T, as shown in Figure 5.

The results illustrated in Figure 5 emphasize the two-state behavior. For A30P, there is even a third state below 1 kT. By definition, the derivative of the curves represented in Figure 5 represents the Density Of conformations or micro-States (DOS). The nearly linear behavior of the curves for WT and E46K up to 2 kT means a rather constant DOS corresponding to state B (one-dimensional probability densities in Figure 4). The change of slope at 2 kT for these proteins points up the separation between the states, i.e., the onset of state HB, i.e., a state with a mixture of *α*-helices and *β*-sheets. State B represents thus 40% of the conformations for WT and E46K. Between 2 kT and about 3 kT, the curves for WT and E46K in Figure 5 are linear with a slope larger than that in the B state, which corresponds to a larger DOS in state HB. Beyond 3 kT, the DOS decreases and reaches a plateau for all proteins. For A53T, state B contains less conformations and the onset of the state HB occurs at about 1.5 kT. State B represents only about 25% of the conformations for A53T. The DOS of the HB state for A53T is higher than that in its B state, and it is also higher than the DOS of the HB state for WT and E46K. The case of A30P is special. State B has the highest DOS up to 1 kT, and it becomes similar to the DOS for WT and E46K up to 2.5 kT. The third state below 1 kT is clearly visible as a shoulder with a large proportion of conformations with 60—70 residues in the *β*-sheet in the one-dimensional probability distribution, shown in Figure 4. Finally, state B in A30P also concerns 40% of the structures. The DOS in the HB state for A30P is similar to the one of A53T.

**FIGURE 6:**
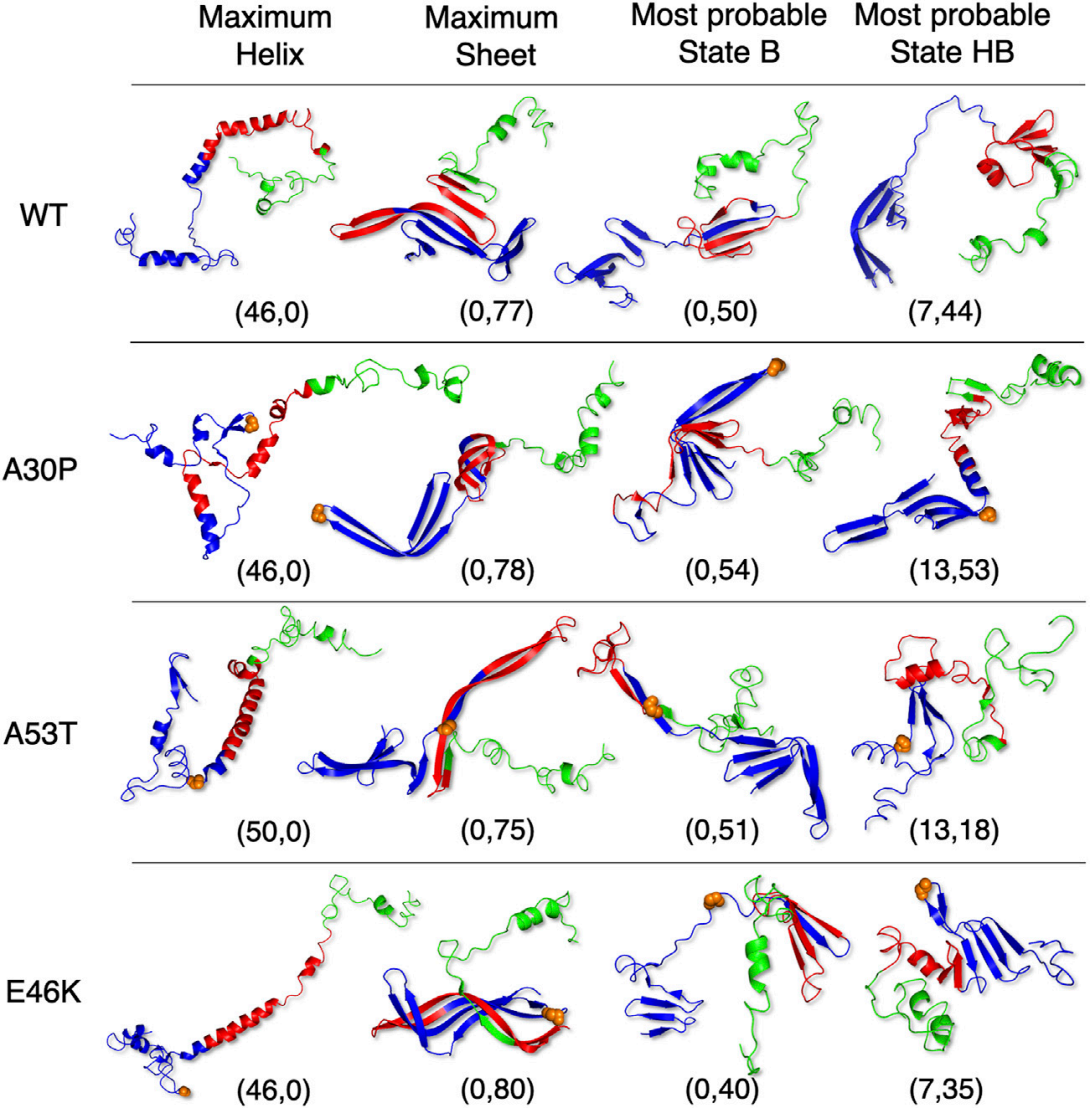
Selected representative structures for WT and mutants extracted from the conformational sub-ensembles corresponding to the maximum fraction in the helix (first column), the maximum fraction in the *β*-sheet (second column), the most probable sub-state of state B (third column), and the most probable sub-state of state HB (last column). The coordinates correspond to the position in (*α*, *β*) maps of Figure 4. Orange spheres represent backbone atoms at the mutation location. The 3D representations were made with the PyMOL software (Schrödinger, 2015).

Figure 5 shows that state HB encompasses about 60% of the conformations for WT, A30P, and E46K (all conformations beyond 2 kT for WT and E46K and beyond 2.5 kT for A30P) and 75% of the conformations for A53T (all conformations beyond 1.5 kT). Although the DOS is relatively constant, local maxima occur in the HB two-dimensional maps (Figure 4). The most probable (*α*, *β*) conformations occur at (7,44), (13,53), (13,18), and (7,35) for WT, A30P, A53T, and E46K. There is a significant difference between A53T and WT, A30P, and E46K. For each protein, selected structures for the maximum of probability of the B state and for the local maxima of the probability of the HB state are represented in Figure 6. They illustrate the expected diversity of conformations of an IDP. It is, however, important to emphasize that each (*α*, *β*) pair represents an ensemble of conformations. It is impossible to represent the entire diversity of these sub-ensembles. For example, for the WT protein, the maximum of the B state at (0,50) corresponds to 19,901 structures, and the maximum of the HB state at (7,44) corresponds to 3,569 structures. In Figure 6, the structures with a maximum of residues in the helix or in the *β*-sheet are shown. The maximum number of residues in the helix is 50 for A53T compared to 46 for the other proteins and compared to the 82 (Ulmer et al., 2005) and 97 residues (Cheng et al., 2013) in helices when *α*-syn is bound to membranes.

The average radius of gyration *R*_*g*_ is a common global structural parameter in polymer science, and it is interesting to relate this property to states B and HB. For a random coil represented by a self-avoiding walk in a good solvent (i.e., for which interactions between monomers and solvent molecules are energetically favorable), *R*_*g*_ = 0.367*bN^ν^*, where *b* is the length of the so-called statistical segment and *ν* is a fractal exponent. In three dimensions, we take *b* = 7.6 Å (twice the distance between two *C^α^*), *R*_*g*_ = 54 Å for the size of *α*-syn (*N* = 140) (Victor et al., 1994). The average radius of gyration *R*_*g*_ computed from the UNRES trajectories is 24.7, 25.1, 26.0, and 25.2 Å for WT, A30P, A53T, and E46K, respectively. Only A53T has a significantly larger *R*_*g*_ than WT. Other authors reported an average radius of gyration of *R*_*g*_ ≃ 23 Å for WT and a distribution of *R*_*g*_ narrower than the distribution of a random coil of a similar sequence length (Dedmon et al., 2005; Allison et al., 2009).

**FIGURE 7:**
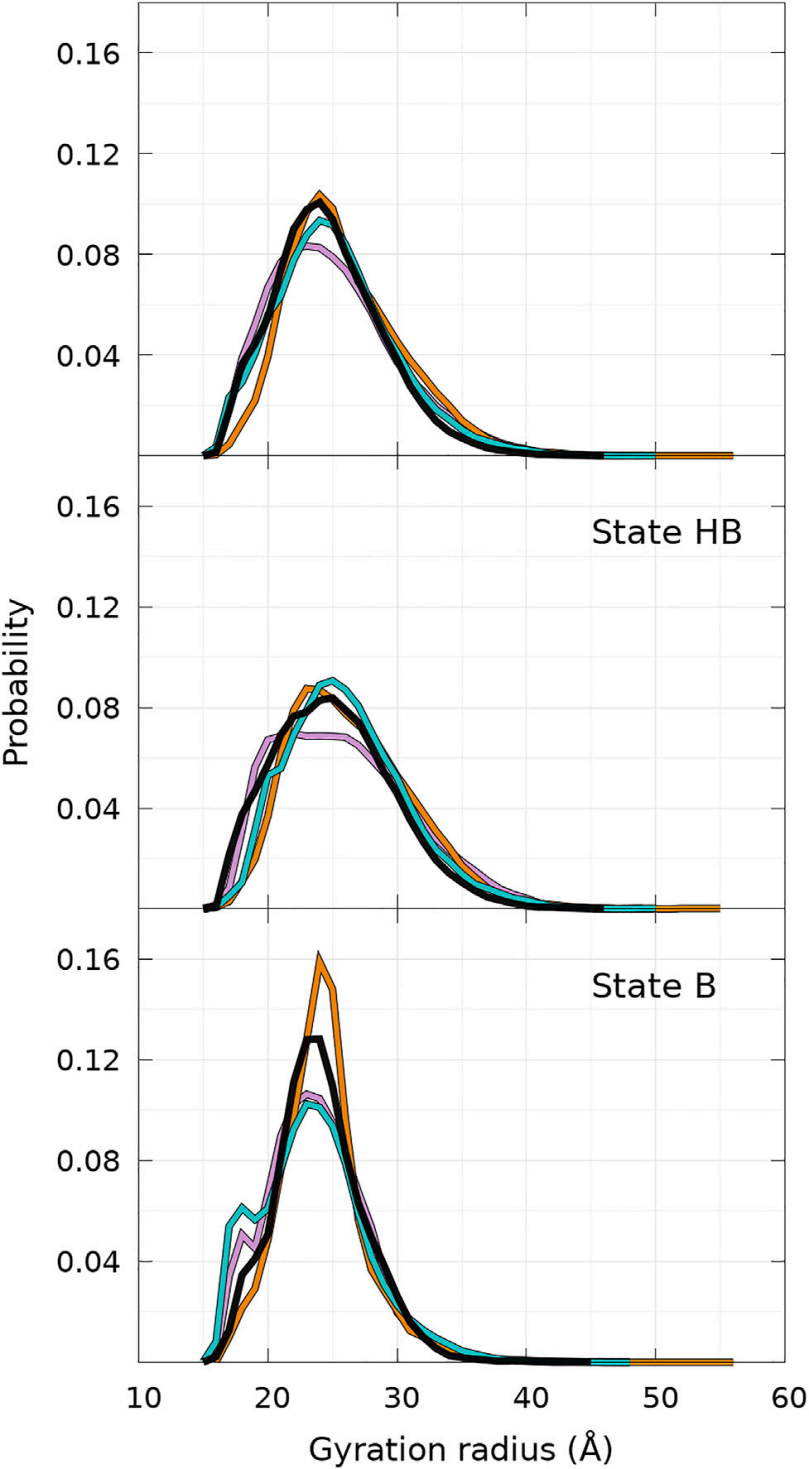
Probability density of gyration radius computed for WT and mutants of *α*-syn for the complete conformational ensemble **(top panel),** the ensemble of state HB **(middle panel),** and the ensemble of state B **(bottom panel)**. The color code is WT (black), A30P (purple), E46K (turquoise), and A53T (orange). State B represents 40% of conformations for WT, A30P, and E46K and 25% for A53T (see text).

The distributions of *R*_*g*_ of the conformational ensemble simulated with UNRES for WT and mutants are very similar to each other, as shown in Figure 7. The peak of the probability density is lower for A30P. When the global distribution is divided into states B and HB, one reaches the same conclusion for state HB (Figure 7, middle panel), but one observes more significant differences between the proteins for state B (Figure 7, bottom panel). For state B, the peak of A53T is the highest and a sub-population appears clearly on the left side of the distribution for A30P and E46K. This sub-population is hardly visible in the global probability distribution (Figure 7, top panel) as a shoulder. Examination of the structures corresponding to this sub-state of the B state reveals that the structures of A30P and E46K have a large proportion of contacts between regions 1—20 and 96—140. The average number of contacts of the structures with 17.9Å < *R*_*g*_ < 18.1 Å is 5.2, 14.9, 0.1, and 9.0 for WT, A30P, A53T, and E46K, respectively. The large number of contacts between the two extremities of the protein for A30P and E46K mutants compared to WT and A53T explains the peak at 18 Å.

### 3.2 Secondary Structure Element Propensities as a Function of the Position in the Amino-Acid Sequence: Differences and Similarities Between the Wild Type and Mutants

**FIGURE 8:**
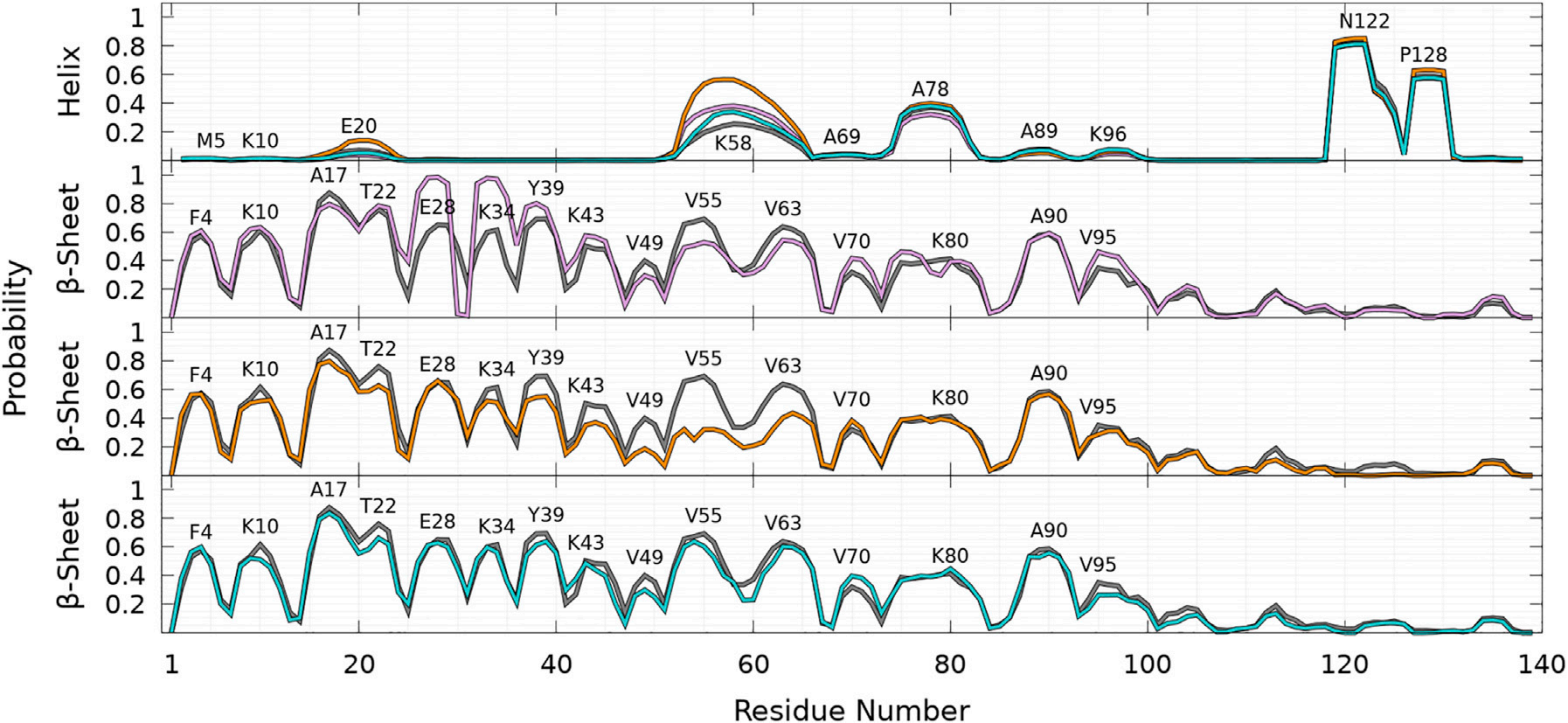
Probability of each residue to pertain to an *α*-helix **(top panel)** and a *β*-sheet (other panels) for WT (gray), A30P (purple), A53T (orange), and E46K (turquoise) as a function of the residue position in the sequence. For the *β*-sheet, each mutant is compared to WT (gray) with labels pointing out residues at local maxima of the WT probability densities.

As shown in Figure 8, helices are found in four main regions: two in the C-terminal region (residues 119—125 and 127—130), one in the NAC region (residues 75—82), and one overlapping the N-terminal and NAC regions (residues 53—65) for both WT and mutants. As claimed in the previous section, there are no significant differences between the propensities of SSE for WT and mutants in the C-terminal region. The major differences between WT and mutants occur in the region 53—65, which has a peak for the helix propensity at residue K58. For all mutants, the probability to form an *α*-helix in this region is larger than that for WT. For A53T, the probability of residue K58 to pertain to a helix is more than twice higher than one for the WT protein (*P*_*WT*_ = 0.25 compared to *P*_*A*53*T*_ = 0.56). Mutation 53 occurs in the N-terminal part of a helical region of WT, but its effect is not trivial as the mutation could be naively expected to decrease the helicity. Indeed, the propensity to be part of a helix is 1.45 for alanine and 0.82 for threonine according to the empirical helix propensity scale (Chou and Fasman, 1974). The increase of helicity observed in the 53—65 region of the sequence upon single mutation is clearly not a local effect. Similarly, single amino-acid substitutions, A30P and E46K, also increase significantly the helicity in the region of 53—65.

Compared to other proteins, another significant difference is observed for A53T in the helical region located at residues 18—22, where the probability to form an *α*-helix is significantly larger for this mutant. For example, the peak at E20 corresponds to the following probabilities: *P*_*WT*_ = 0.07, *P*_*A*30*P*_ = 0.04, *P*_*A*53*T*_ = 0.14, and *P*_*E*46*K*_ = 0.05. The significant increase of helicity in the 18—22 and 53—65 regions explains why state B of A53T is less populated, as shown in Figures 4, 5 and discussed in the previous section. The presence of an *α*-helix in the 18—22 region might explain why mutation A18T induces significant modification of the *α*-syn polymerization (Kumar et al., 2018). Simulations of this mutant are scheduled in the future. For A30P, the probability to observe a helix in the NAC region is slightly lower than for other proteins: the probabilities at A78 are *P*_*WT*_ = 0.37, *P*_*A*30*P*_ = 0.32, *P*_*A*53*T*_ = 0.40, and *P*_*E*46*K*_ = 0.38.

As shown in Figure 8, the probabilities to find residues in the *β*-sheet are significantly high in the N-terminal and NAC regions up to about residue 100 at the same locations for WT and mutants. For WT, the maximum of the peaks observed in Figure 8 are *P*_*F*4_ = 0.57, *P*_*K*10_ = 0.61, *P*_*A*17_ = 0.87, *P*_*T*22_ = 0.76, *P*_*E*28_ = 0.65, *P*_*K*34_ = 0.61, *P*_*Y*39_ = 0.69, *P*_*K*43_ = 0.50, *P*_*V*49_ = 0.4, *P*_*V*55_ = 0.69, *P*_*V*63_ = 0.64, *P*_*V*70_ = 0.32, *P*_*K*80_ = 0.41, *P*_*A*90_ = 0.58, and *P*_*V*95_ = 0.35.

As shown in Figure 8, the *β*-sheet probabilities are very similar for WT and E46K along the sequence and differ significantly for A30P in the region 26—80 and for A53T in the region 35—65. The mutation A30P has a huge local impact on the probability of residue 30 to pertain to a *β*-sheet, *P*_*WT*,*A*30_ = 0.49 and *P*_*P*30_ = 0.03. This induces an unexpected increase of the probability to occur in the *β*-sheet for the neighboring residues: *P* = 0.85 for residues 26—29 and 32—35. Long-range effects of A30P mutation on the propensities of other residues are observed by an increase of the peaks at *P*_*Y*39_ = 0.76, *P*_*K*43_ = 0.57, *P*_*V*70_ = 0.42, *P*_*T*75_ = 0.46, and *P*_*V*95_ = 0.46 and by a decrease of the peaks at *P*_*V*55_ = 0.53 and *P*_*V*63_ = 0.54, compared to WT. The decrease in *β*-sheet propensity in this region compared to WT agrees with their larger helical propensity in this region for the A30P mutant (Figure 8). For A53T, the most drastic effect of the single amino-acid substitution occurs in the region 53—65, where the probability to form *β*-sheets is significantly reduced compared to WT, *P*_*V*55_ = 0.32 and *P*_*V*63_ = 0.40, in agreement with their high probabilities to be in a helix (Figure 8). Other significant long-range effects of the amino-acid substitution are observed at peaks *P*_*Y*39_ = 0.55, *P*_*T*44_ = 0.37, and *P*_*V*49_ = 0.18, where the mutation A53T decreases the probability to form a *β*-sheet compared to WT. Overall, two crucial regions of the amino-acid sequence are affected by the mutations: the region 26—35, where the A30P mutation increases mainly the *β*-sheet formation, and the region 53—65, where the A53T mutation mainly decreases the *β*-sheet formation.

**FIGURE 9:**
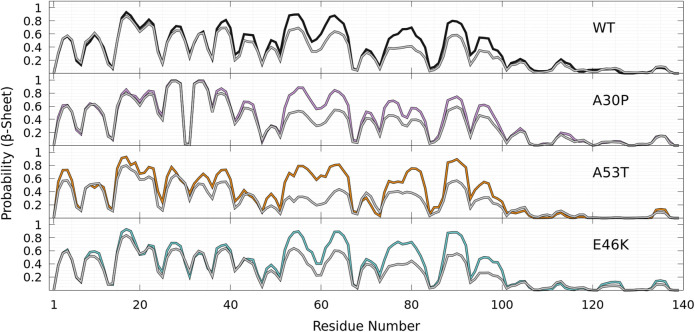
Probability of each residue to pertain to a *β*-sheet for WT (black), A30P (purple), E46K (turquoise), and A53T (orange) in the B state compared to the probability in the corresponding entire (B + HB) ensemble of conformations for each protein (gray) as a function of the residue position in the sequence.

Finally, as shown in Figure 9 for all proteins, the statistics of *β*-sheet propensity of state B differs from the global statistics (B + HB) in the region 50—100, which encompasses the two major helical regions centered at K58 and A78 (Figure 9, top panel). The most significant differences are observed for A53T, for which the probability to form a *β*-sheet is significantly lower in the HB state in the region 50—70, where the probability to form an helix is very high (Figure 9).

## 4 Discussion

The present analysis is based on a first-principle (no bias or constraints applied) very large conformational sampling of the WT *α*-syn and mutants. As in any MD simulations, the sampling is never complete and each force-field has some bias. Next, we compare the predictions of UNRES for WT and mutants to available experimental data and previous theoretical studies.

**FIGURE 10:**
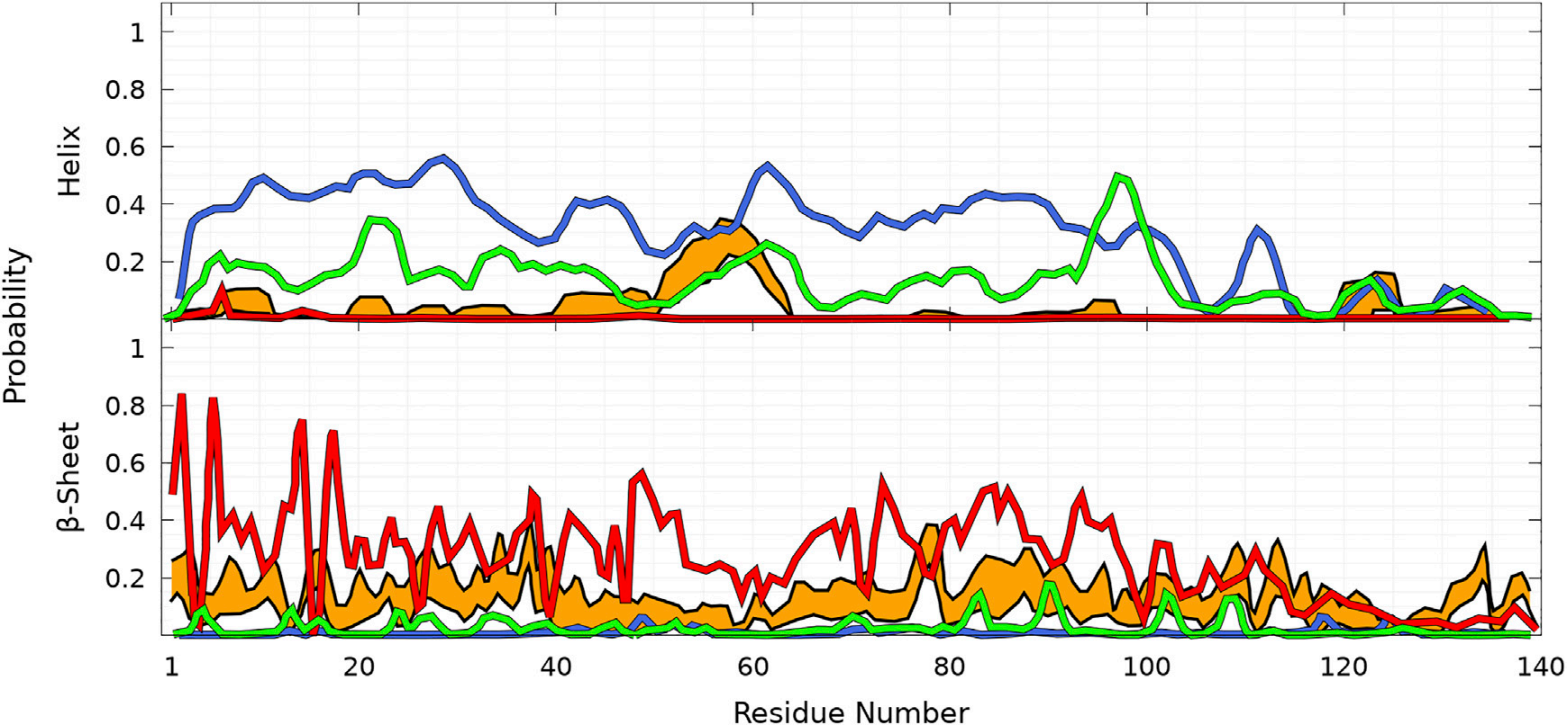
Probability of each residue of WT to pertain to an *α*-helix **(top panel)** and a *β*-sheet **(bottom panel)** as a function of its position in the sequence from MD simulations according to four different works: Ullman et al., 2011 (orange), Yu et al., 2015 (blue), Ramis et al., 2019 (red), and Coskuner and Wise-Scira, 2013 (green). The thickness of the orange lines corresponds to the 95% confidence interval. Excerpted of the published figures of the works cited are reproduced with permissions. Further permissions related to the material excerpted should be directed to the corresponding publishers.

To illustrate the difficulties of producing a conformational ensemble of *α*-syn, we compared (see Figure 10) the helix and *β*-sheet probabilities computed in four different works using MD simulations for WT *α*-syn (Ullman et al., 2011; Yu et al., 2015; Ramis et al., 2019; Coskuner and Wise-Scira, 2013). Compared to the UNRES simulations and Ref. Ullman et al., 2011, the calculations of Ref. Yu et al., 2015 largely overestimate the helical properties of *α*-syn, whereas the simulations of Ref. Ramis et al., 2019 largely underestimate them. The simulations of Ref. Coskuner and Wise-Scira, 2013 also predict a larger helical propensity than the one found in the present work (Figure 8) and in Ref. Ullman et al., 2011 (Figure 10). It is worth noting that the helical region nearby residue 60 is found in the UNRES simulations (Figure 8) and in Refs. Ullman et al., 2011; Coskuner and Wise-Scira, 2013 (Figure 10).

Comparison with our predictions shown in Figure 8 indicates better agreement with Ref. Ullman et al., 2011 for the helical part, except that UNRES predicts a larger probability to form an *α*-helix at residue A78. In the present work, MD simulations are performed for the entire protein. In contrast, the method of Ref. Ullman et al., 2011 consists of a combination of MD simulations of peptides of *α*-syn with sequences which overlap each other and are extracted from the *α*-syn sequence. The conformation of the protein is rebuilt by a combination of these fragments with weights using NMR data. The formation of transient helical segments was observed experimentally in the WT *α*-syn tetramer in Ref. Wang et al., 2011 between residues 4—43 and 50—103. Larger probabilities of helical tendency were found experimentally in regions 20—23 and 48—90 (Wang et al., 2011). These findings are rather in good agreement with the transient existence of helices in the *α*-syn monomer, as shown in Figure 8 (top panel), including the peak at A78. The stability of the tetrameric form is supported by previous all-atom MD simulations showing that the stability of this helical oligomer is due to a hydrophobic core formed by non-polar residues pertaining to the second *α*-helix of each chain, along with salt bridges formed by positively charged lysine residues and negatively charged aspartate and glutamate residues (Cote et al., 2018).

A detailed quantitative comparison between the UNRES *β*-sheet propensities (Figure 8) and those predicted in Refs. Ullman et al., 2011; Coskuner and Wise-Scira, 2013; Yu et al., 2015; Ramis et al., 2019 is difficult. However, we observe that there is a significant tendency to form *β*-sheet conformations over the entire sequence in Refs. Ullman et al., 2011; Ramis et al., 2019. On the contrary, simulations of Refs. Coskuner and Wise-Scira, 2013; Yu et al., 2015 predicted much localized and lower *β*-sheet probabilities.

Although the probabilities represented in Figure 8 for intra-molecular *β*-sheets of an isolated monomer cannot be simply compared to the formation of intra-molecular contacts in fibrils, the peaks in UNRES predictions for the *β*-sheet propensities (Figure 8) agree with the pairs of residues forming intra-molecular contacts in protofilaments, i.e., 47–79, 48—78, 92—71, 93—70, 94—69, 95—68 (PDB ID: 2n0a), and 47—79, 48—78, 92—69, 93—68 (PDB ID 6h6b). In addition, the maxima in Figure 8 for *β*-sheet propensity are located at or close to valine residues [*P*_*V*3_ = 0.52, *P*_*V*26_ = 0.46, *P*_*V*40_ = 0.57, *P*_*V*49_ = 0.4, *P*_*V*55_ = 0.69, *P*_*V*63_ = 0.64, *P*_*V*70_ = 0.32, *P*_*V*82_ = 0.31, *P*_*V*95_ = 0.35], which is the most frequently found amino acid in *β*-sheets (Chou and Fasman, 1974). The peak at A90 is in the hydrophobic stretch 88IAAA91. Present simulations show a significant propensity to form a *β*-sheet in the region 71—83 necessary for aggregation (Giasson et al., 2001). Except for G73, the probability is indeed about 40% in this region (Figure 8, bottom panel). However, as mentioned above, in the present simulations, the probability of helical tendency at A78 is also about 40%. The results shown in Figure 8 are thus compatible with the probability to form both the *β*-sheet and helix in the region 71—83. The helix can be stabilized in the tetrameric oligomer (Bartels et al., 2011; Wang et al., 2011; Cote et al., 2018).

Although the propensities along the sequence are difficult to compare to experimental data, more global metrics can be used. Indeed, an interesting experimental parameter is the average content in helix and *β*-sheet conformations of WT *α*-syn in solution. Circular dichroism (CD) data reported an average of 2 ± 3% and 11 ± 7% for helix and *β*-sheet contents, respectively (Rekas et al., 2010). It is worth noting that the algorithms to extract *β*-sheet conformations from CD spectra are not as accurate as those for helices (Micsonai et al., 2015) and, as shown for *α*-syn, the CD spectrum is also dependent on the buffer and concentration (Araki et al., 2016). Other experimental values extracted from CD for the SSE content of WT monomers were reported: < 2% for the helix and 30% for *β*-sheet contents in Ref. Weinreb et al., 1996 and 3 ± 1% for the helix and 23 ± 8% for *β*-sheet fractions in Ref. Davidson et al., 1998. In their construction of the WT conformational ensemble based on MD simulations of *α*-syn fragments using the CHARMM force field constrained by NMR data, Ullman et al. reported the values 2% for the helix and 11% for *β*-sheet fractions (Ullman et al., 2011) (orange curve in Figure 10). In the present work, the global proportions of residues in the helix and *β*-sheet computed from the complete converged conformational WT ensemble are 10 and 31%, respectively. There are about 3 times more residues in the *β*-sheet than in the helix, a ratio which is in agreement with the one estimated from CD in Ref. Rekas et al., 2010. Interestingly, the theoretical values reported by coarse-grained simulations of WT *α*-syn with a different force field (red curve in Figure 10) are 20 ± 4% and 26.8 ± 6.8% for helix and *β*-sheet fractions, respectively (Ramis et al., 2019). In the present simulations, one notes that half of the helix fraction arises from the short helices located in the C-terminal region. The helix fraction without the C-terminal region is only 5% here. The fractions of the helix and *β*-sheet are on the same order of magnitude for the mutants and WT: A30P (helix = 10%, *β*-sheet = 34%), A53T (helix = 13%, *β*-sheet = 26%), and E46K (helix = 10%, *β*-sheet = 29%). Again, A53T shows a larger deviation compared to WT in agreement with a less populated B state (Figures 4 and 5). Most likely, the present force field overestimates the formation of SSE but predicts the correct equilibrium between the two main SSEs.

Another global structural parameter is *R*_*g*_. The average radius of gyration *R*_*g*_ measured by small-angle X-ray scattering for WT *α*-syn in solution depends on the protein concentration, the presence of dimers or trimers, buffer type, pH, acetylation, and the source of proteins (Araki et al., 2016). For recombinant *α*-syn in 10 mM ammonium acetate pH 7.4, *R*_*g*_ = 27.2 ± 0.44 Å extrapolated at infinite dilution, which is comparable to the average computed value (*R*_*g*_ = 24.7 Å, Figure 7). Addition of HCl and/or NaCl increases *R*_*g*_ significantly to 33—40 Å (Li et al., 2002; Allison et al., 2009; Araki et al., 2016), but these effects cannot be tested with the present model. It is interesting to note that *R*_*g*_ for dimers in the present simulations are about 10 Å larger than that of monomers, as will be discussed elsewhere. Therefore, any mixture of monomers and dimers increases the effective radius of gyration of the solution.

The effects of the missense mutations on the structural properties can be summarized as follows: for the SSE tendency (Figure 8), the most significant effects of the single mutations are for A30P and A53T. Residue P30 has a huge effect on neighboring residues for the formation of the *β*-sheet. Residue T53 has a great influence on the helical region centered at K58. For the B state, structures with low *R*_*g*_ (around 18 Å) are more compact for A30P and E46K due to the large average number of contacts between the residues 1—20 and 96—140. For the entire ensemble of conformations, the average number of contacts between these two regions is rather similar for WT (1.84), A30P (2.12), and E46K (1.61) but significantly less for A53T (0.75). The mutant A53T is thus expected to be more flexible and less compact as shown by its larger *R*_*g*_. The analysis is slightly different if the average number of contacts is computed between the entire N-terminal (1—60) and C-terminal (96—140) regions for which the average number of contacts is 4.54 (WT), 4.22 (A30P), 2.71 (E46K), and 2.61 (A53T). One finds that both E46K and A53T are much more flexible than WT. NMR studies of mutants A30P and A53T showed a reduction of contacts between C- and N-terminals for both mutants compared to WT (Bertoncini et al., 2005). The present MD results fully agree with these data for A53T.

A major finding in the present work is that an isolated *α*-synuclein in solution occurs in two phases, which are clearly visible in Figures 4 and 5. Unfortunately, it is difficult to construct experimentally a two-dimensional map of SSE propensities such as in Figure 4. A possible road toward such experimental analysis could be the use of Raman single-molecule spectroscopy (Leray et al., 2016; Dai et al., 2021) as *α*-helices and *β*-sheets have been well described by Raman fingerprints (Dai et al., 2021). About 40% of conformations are in the B state for WT, A30P, and E46K and 25% for A53T, and such a significant fraction of the conformational ensemble might be detectable by spectroscopy.

## Data Availability

The original contributions presented in the study are included in the article/Supplementary Materials, and further inquiries can be directed to the corresponding author.
