# Supplementary material for: Missense Mutations Modify the Conformational Ensemble of the α-Synuclein Monomer Which Exhibits a Two-Phase Characteristic
Source: Front Mol Biosci. 2021 Nov 29;8:786123. doi: 10.3389/fmolb.2021.786123 (PMC8667727; doi:10.3389/fmolb.2021.786123)
Supplement: Supplementary file 1 [file Image1.pdf]

Supplementary Material

1 SUPPLEMENTARY FIGURE

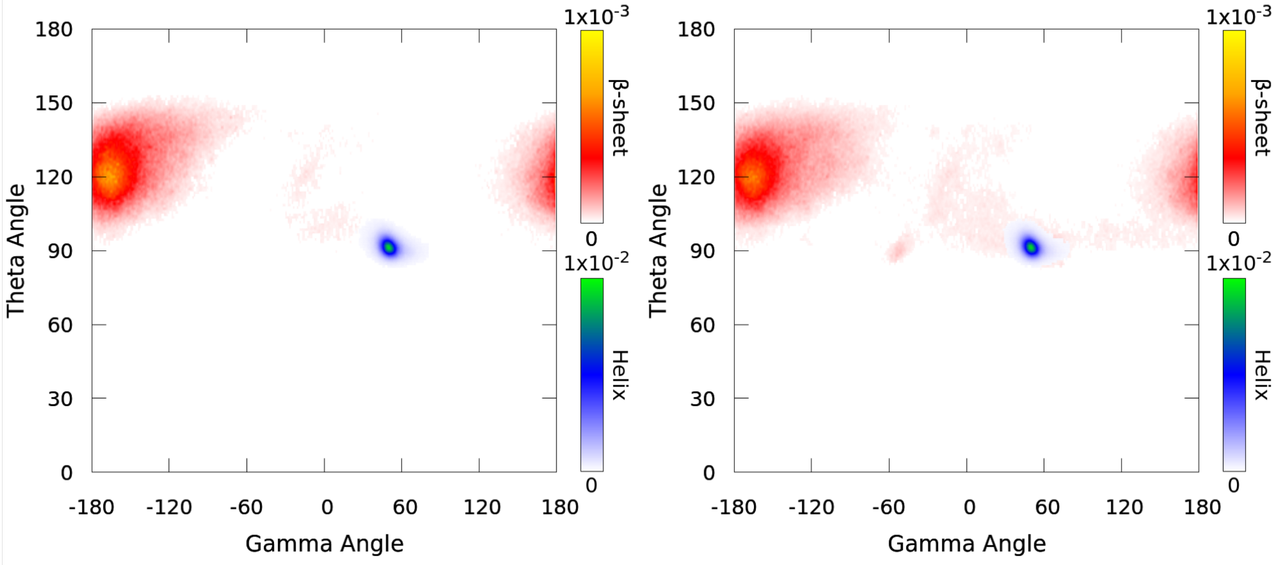

Figure S1. Probability density of α helix (blue color scale) and β-sheet (red color scale) as function of the coarse-grained angles θ and γ. The maps were computed for all the structures in the ASTRAL database of proteins with less than 40 % of sequence identity. Calculations based on secondary structure identifications by the DSSP algorithm are shown in the left panel and those based on the CUTABI algorithm are represented in the right panel.
